# Presurgical Rehearsals for Patients Considering “Awake” Deep Brain Stimulation

**DOI:** 10.3389/fsurg.2016.00044

**Published:** 2016-08-02

**Authors:** Ramsey A. Falconer, Sean L. Rogers, Cristie M. Brewer, Franco Piscitani, Mahesh B. Shenai

**Affiliations:** ^1^Department of Neurology, Inova Neuroscience and Spine Institute, Falls Church, VA, USA; ^2^Department of Neurosurgery, Inova Neuroscience and Spine Institute, Falls Church, VA, USA; ^3^Advanced Surgical Technology and Education Center, Inova Fairfax Hospital, Falls Church, VA, USA

**Keywords:** DBS, surgical simulation, Parkinson’s disease, awake neurosurgery, patient experience

## Abstract

Simulated surgical environments are rapidly gaining adoption in training students, residents, and members of specialized surgical teams. However, minimal attention has been given to the use of simulated surgical environments to educate patients on surgical processes, particularly procedures that require the active participation of the patient. “Awake” neurosurgery provides a unique situation in which patients openly participate in their operation. We describe a case report, in which a 62-year-old male was referred for “awake” deep brain stimulation implantation, in relation to medically refractory Parkinson’s disease. The patient had significant concerns regarding anxiety and claustrophobia, and toleration of the “awake” procedure. Consequently, we designed a simulated OR environment and process, to recreate the physical experience of the procedure, with minimal cost or risk. This experience was crucial in determining the care plan, as after this experience, the patient opted for an “asleep” alternative. Thus, in certain settings, presurgical rehearsals may have a dramatic impact in the overall course of care.

## Introduction

Simulated surgical environments are rapidly gaining adoption in training students, residents, and members of specialized surgical teams (1–3). Recreating surgical circumstances allows for participants to rehearse roles and duties, with evidence pointing toward superior performance and outcomes in real settings (4–6). However, minimal attention has been given to the use of simulated surgical environments to educate patients on proposed surgical processes or procedures, a notion that would potentially lead toward increased patient empowerment and satisfaction. “Awake” neurosurgery provides a unique situation in which patients openly participate in their operation (7), and thereby, may benefit from a preoperative rehearsal.

Deep brain stimulation (DBS) is a remarkable therapy, which is approved for the treatment of Parkinson’s disease (PD), essential tremor (ET), and dystonia (8). Historically, the vast majority of DBS implantations are performed “awake” (or with minimal sedation), in order to record neurophysiology and test for stimulation effect. This “awake” testing has been critical in obtaining optimal outcomes, as the presence of adverse effects to stimulation could compel intraoperative adjustment of the lead positioning.

However, the “awake” procedure itself requires significant patient endurance and maturity, which naturally creates a number of eligible patients who defer its dramatic benefit due to the prospective stress of an “awake” procedure. Recently, “asleep” DBS implantation has gained traction, allowing a neurosurgeon to place DBS electrodes under general anesthesia with the use of intraoperative MRI to guide anatomic placement of the stimulation lead. While potentially more tolerable to the patient, the lack of real-time feedback could result in a functionally suboptimal placement. To date, there is no evidence that “asleep” DBS implantation is superior to “awake” implantation, but the emerging consensus suggest comparable outcomes (9).

During preoperative counseling, the neurosurgeon may present the patient with either the “awake” or “asleep” implantation option, along with the respective benefits and drawbacks of each technique. However, from the patient’s perspective, verbal or visual descriptions of the “awake” procedure insufficiently describe the true surgical experience. Therefore, a preoperative surgical rehearsal could better mimic the true experience, and help the patient choose between the “asleep” or “awake” procedure, and improve participation if the latter is chosen. In this case report, we present a patient who was concerned about the awake procedure, so was given a surgical rehearsal that provided critical guidance on his care pathway.

## Case Report

G.B. is a 62-year-old male with a 7-year history of right-sided PD symptoms, including bradykinesia, rigidity, and a resting tremor. He was officially diagnosed with PD 5 years prior after consultation with a movement disorder specialist. In the initial phase of his treatment, G.B. responded quite well to standard l-DOPA therapy, as is typically the case with idiopathic PD. However, as the course of his disease progressed, the dosage and frequency of l-DOPA had to be increased to maintain therapeutic effect, eventually leading to troublesome breakthrough symptoms and dyskinesia. Socially, G.B. is extremely high-functioning, with a college degree, and employed as a caretaker in a nursing facility. In December 2015, G.B. was referred for neurosurgical implantation of DBS.

During neurosurgical consultation, G.B. initially demonstrated enthusiasm for DBS therapy, and strongly met objective criteria for implantation. He had no absolute contraindication to DBS therapy. During the preoperative discussion, the “awake” procedure was described in detail, including (1) placement of a stereotactic head frame, secured by four percutaneous screws placed under local anesthetic, (2) rigid fixation of the head and frame to the bed, limiting movement of the head throughout the procedure, (3) positioning the head and body in a semi-reclined position, (4) an intraoperative CT scan, (5) sterile draping that partially obscures the face and visual fields, (6) incision and transcranial perforation, (7) neurophysiological monitoring, (8) active stimulation testing, and (9) permanent lead implantation. With this description, G.B. expressed his concerns, which included sensitivity to claustrophobia, anxiety, and subjective positional breathing difficulties. Based on these concerns, a surgical rehearsal was suggested, and he agreed to participate.

**Figure 1:**
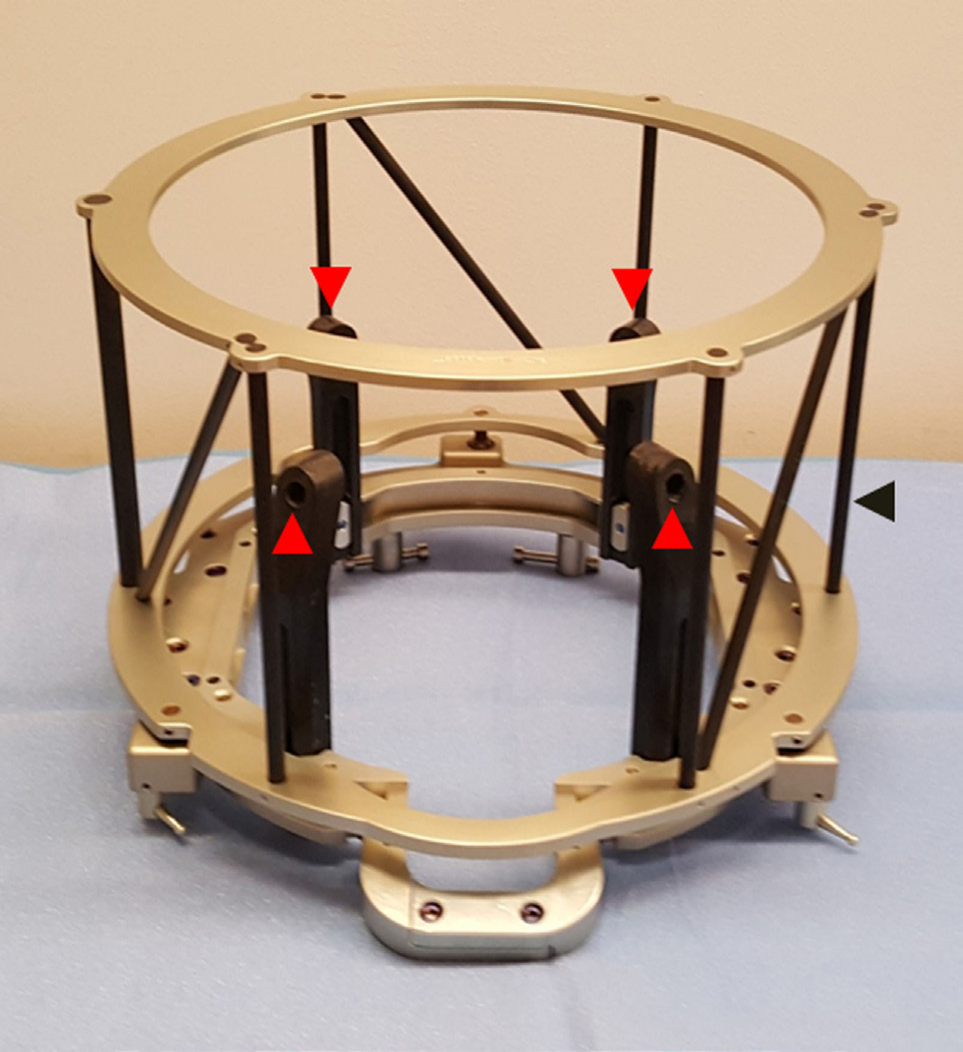
**Cosman–Roberts–Wells (CRW) head frame used for rigid fixation to cranium for stereotactic neurosurgery**. Frame is affixed to cranium via four screws placed through graphite posts (red arrows). Outer cage (black arrow) serves as a CT localizer for image registration.

**Figure 2:**
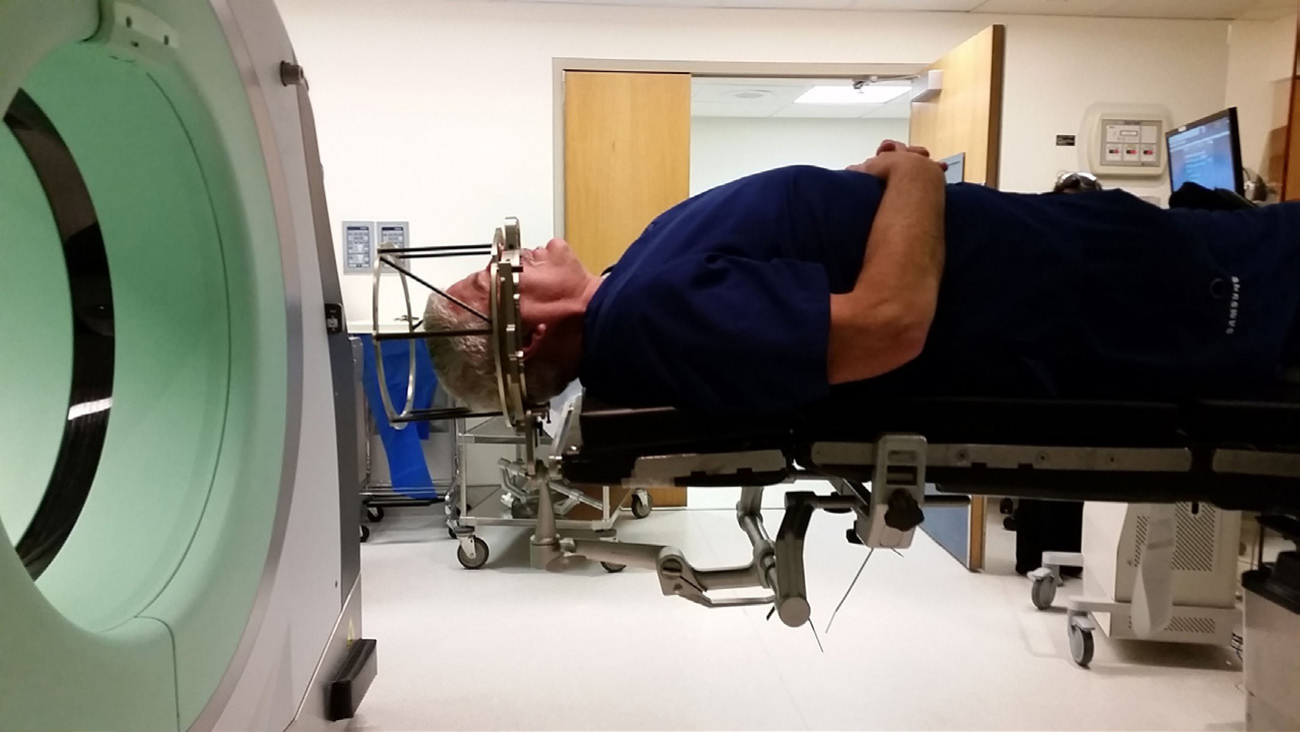
**Positioning of potential patient in CRW frame, prior to CT scanning**.

**Figure 3:**
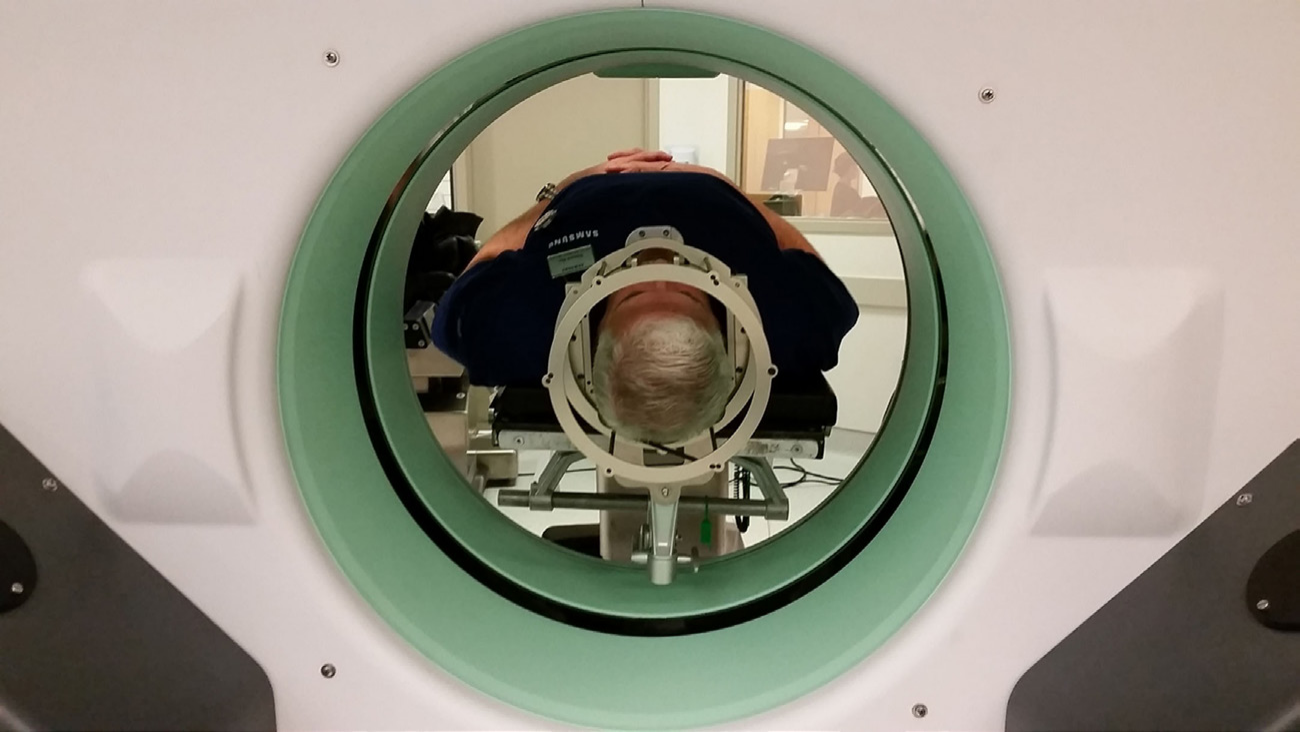
**Simulation of intraoperative CT scanning for image registration and DBS planning**.

**Figure 4:**
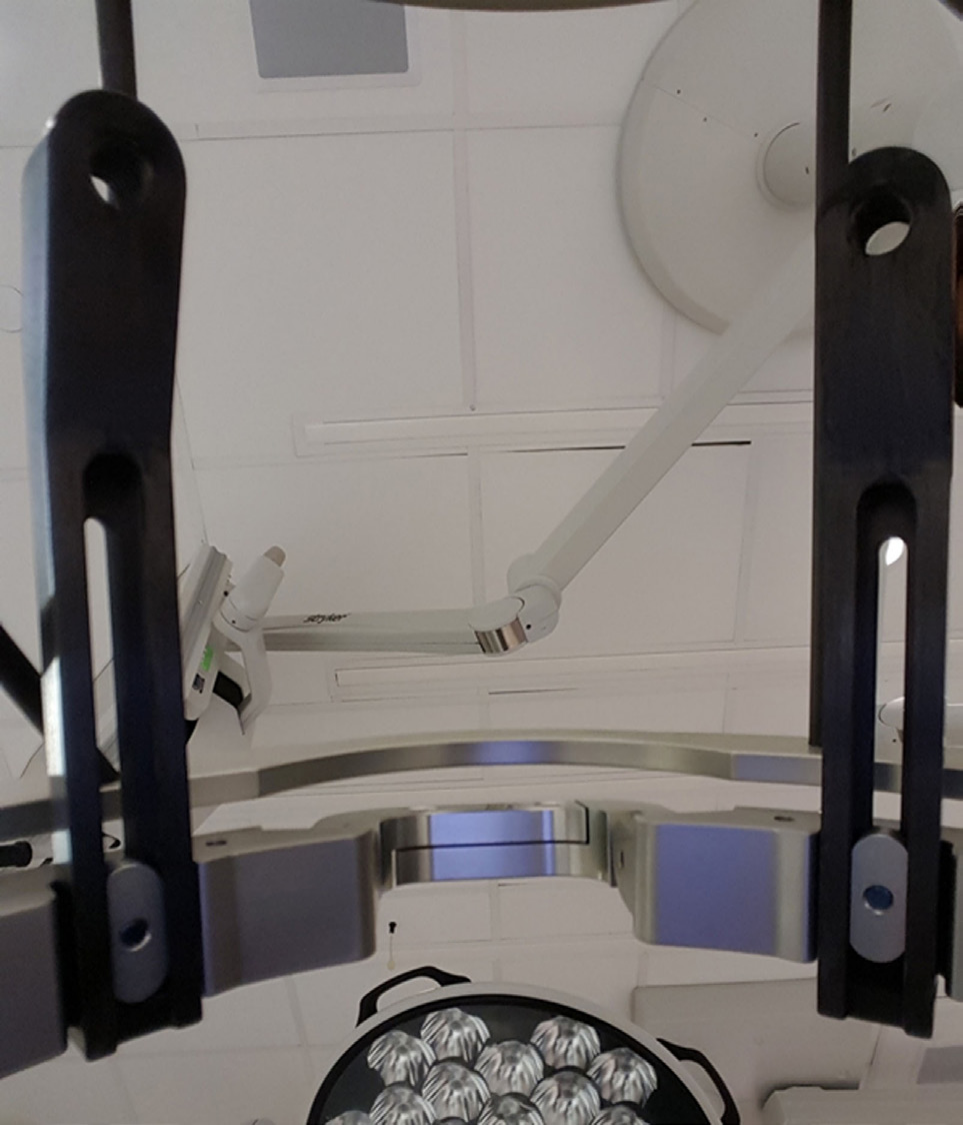
**Simulated patient perspective of presurgical positioning, situated from within the CRW frame, prior to draping**.

**Figure 5:**
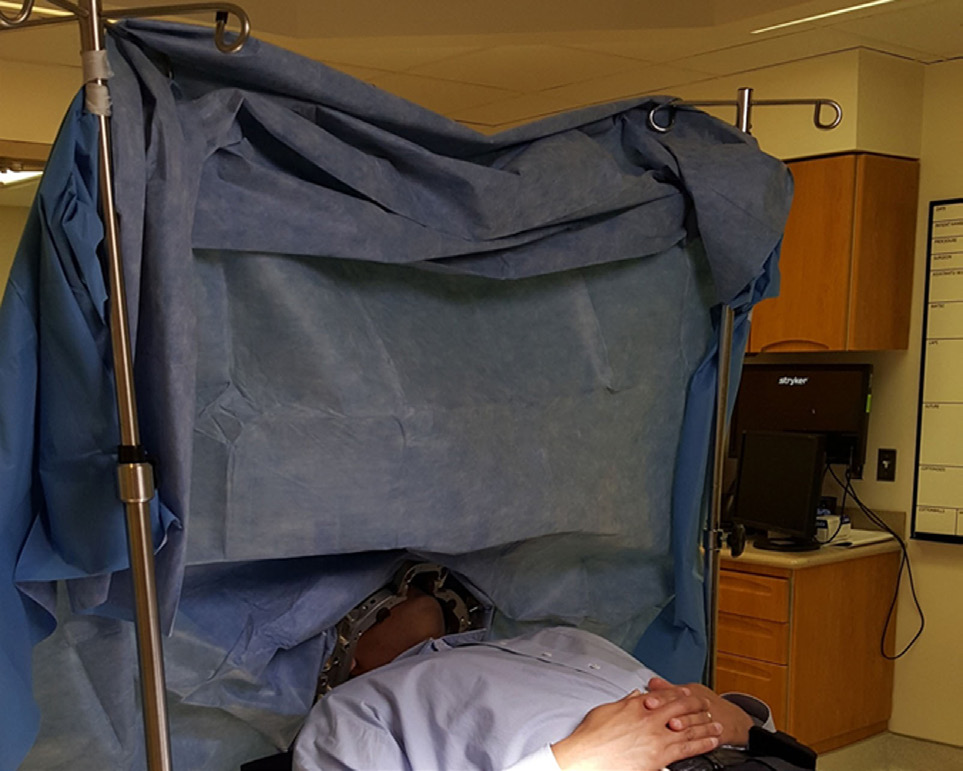
**Surgical positioning after sterile draping**. The sterile field is located behind the drape (not visualized), whereas the interactive patient space is located on the near side of the drape (visualized). This arrangement allows for the team to perform “awake” examination of the arms, legs, and face during deep brain stimulation.

**Figure 6:**
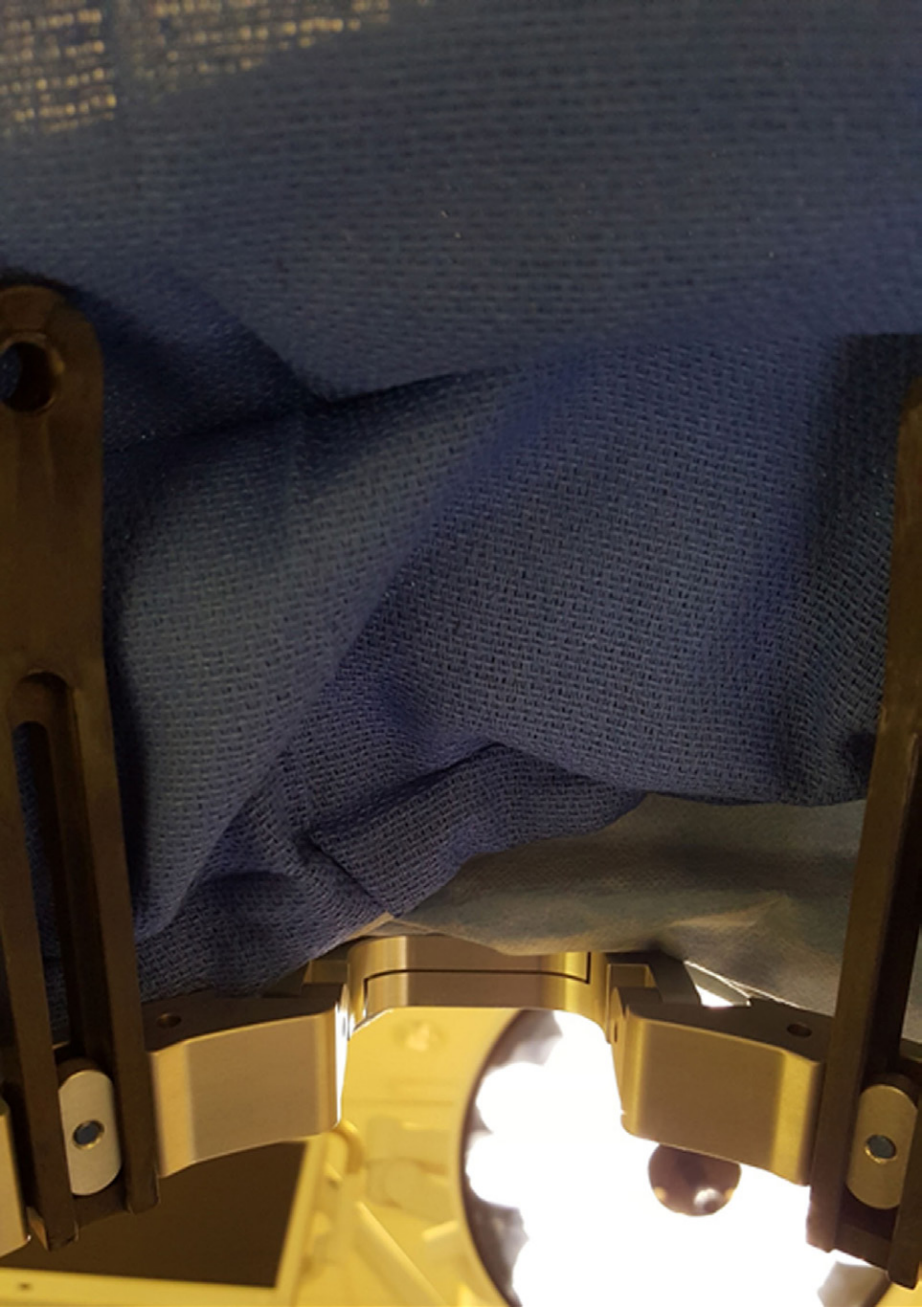
**Simulated patient perspective, after sterile draping, during the DBS procedure**. The patient may experience this viewpoint for 4–6 h, as the surgery is being performed.

Three days later, G.B. and his spouse presented to the Inova ASTEC (Advanced Surgical Technology and Education Center), a 7,000 square foot facility, containing two replica OR suites, a classroom space and other medical simulation facilities. The replica OR was situated in a similar fashion to what would be anticipated in a real DBS implantation. G.B. was brought into the OR, where he was placed on the surgical table. The Cosman–Roberts–Wells (CRW) (Integra, Burlington, MA, USA) rigid stereotactic frame was placed over his head without the percutaneous screw fixation that would be required in the real setting (Figure 1). The patient was then positioned in a supine position, with the CRW frame rigidly fixed to the bed (Figure 2). The intraoperative CT (Bodytom, Samsung) was then maneuvered into place, to provide G.B (Figure 3) with a realistic appreciation of the process. The patient was able to experience a first-person perspective of his rigidly fixed visual field (Figure 4). Once the CT machine was removed, standard sterile prepping and draping was performed (Figure 5), recreating the “awake” spatial environment (Figure 6). Intraoperative neurological testing was practiced to educate G.B. on what would be required of him during the real procedure. The entire rehearsal took ~1 h, with another 30 min dedicated to further discussion with the patient and spouse. All figures presented in this report were recreated for demonstration purposes, as the patient declined to be photographed.

During this rehearsal, it was evident that G.B.’s concerns of tolerating an “awake” procedure were well-founded. He noted significant anxiety even prior to simulated placement of the CRW frame. During the positioning and simulation of the CT scan, he noted even more anxiety, and subjective pharyngeal discomfort that he felt was impacting his breathing. He noted an elevated heart rate as well. During our post-rehearsal discussion, he had a more clear understanding of the process and the reasoning behind each step. While acknowledging the benefits of the “awake” procedure, he acknowledged that his fears and anxieties were irrational, yet significant enough that he would not choose an “awake” procedure. G.B. endorsed a clear desire for the “asleep” procedure, which he ultimately underwent at a later date.

## Discussion

“Awake” brain surgery is a unique situation, one in which the patient serves an active and critical role in the overall outcome of the procedure. As investigators have extensively shown that simulation is valuable to enhance provider performance (5, 6), it is a reasonable hypothesis that “awake” brain surgery patients would also extract benefit from such surgical rehearsals. In the single case presented here, the rehearsal was sufficient in convincing the patient and his spouse (and the surgeon) to pursue an alternative surgical strategy – potentially saving OR time and cost, along with preserving the overall patient experience. In a sense, presurgical rehearsals serve as a type of exposure therapy for patients by utilizing the concepts of exposure and stress inoculation, which have been shown to reduce performance anxiety and improve overall performance (10). As such, it may be possible that a patient is comforted by the rehearsal experience, and thereby selects the “awake” DBS procedure – converse to the result described in this single-patient experience.

While many patients refer to online videos or photographs for education, only a truly high fidelity physical experience can replicate the circumstances affecting the “awake” brain surgery patient. First, the patient learns from a first-person perspective the entire OR orientation (e.g., sterile fields, anesthesia apparatus). Second, the patient experiences the head and neck positioning in relation to the torso, which many patients describe as uncomfortable. The patient can then predict (or at least, anticipate) whether a particular position will be tolerable for long periods of time. Third, the sterile draping process confines the visual fields and physical space around the face and airway producing a challenging obstacle to patients, especially those who may be claustrophobic. In a simulated setting, patients can practice communicating with the OR team to make reasonable countermeasures. Fourth, the surgeon and patient can practice a preemptive dialog (e.g., “You are going to hear the sound of the drill make a hole in your skull.”) or surgical process (e.g., an intraoperative neurological exam, intraoperative CT), that may preempt surprises or anxiety during the real procedure. In general, a presurgical rehearsal may serve as an antidote of exposure and rationalization to counter pre-existing notions and fears.

Presurgical rehearsals, however, do have limitations. For example, the specific circumstance of pain cannot be replicated and, therefore, the rehearsal may induce a sense of patient overconfidence. Similarly, the duration of the actual procedure cannot be efficiently replicated given the time constraints of the patient and surgical team members to participate in the rehearsal. Consequently, patients may underestimate certain circumstances (e.g., positioning, claustrophobia) that could become challenging with longer procedural times. Additionally, many centers do not have a replica OR, and such a presurgical rehearsal would not be feasible, although elements could be performed in any multipurpose room. Finally, unanticipated and unpracticed events could occur during real surgery that could unnecessarily heighten the anxiety in the patient.

Providing preoperative rehearsals do utilize resources that may or may not be available at all institutions. As a real OR is unlikely to be used for simulation due to patient demands, realistic rehearsal requires access to a simulation facility. The salient surgical instruments and apparatuses (e.g., the CRW frame, intraoperative CT) would need to be free of use from other cases. Finally, time is a significant constraint for members of the surgical team who have competing patient care and/or administrative duties. Nevertheless, the ability to provide a presurgical rehearsal for selected patients can have a dramatic impact in the course of care, expectations, and outcomes, as it did for the patient described in this report.

## Author Contributions

RF: patient selection, patient management, and manuscript writing/editing. SR: patient selection, patient management, and manuscript writing/editing. CB: manuscript writing/editing and figures. FP: simulation execution/support and manuscript writing/editing. MS: concept formulation, manuscript writing, editing, and figures.

## Conflict of Interest Statement

The authors declare that the research was conducted in the absence of any commercial or financial relationships that could be construed as a potential conflict of interest.
